# Direct on-Chip Optical Communication between Nano Optoelectronic Devices

**DOI:** 10.1021/acsphotonics.4c01375

**Published:** 2025-01-21

**Authors:** Vidar Flodgren, Abhijit Das, Joachim E. Sestoft, David Alcer, Thomas K. Jensen, Hossein Jeddi, Håkan Pettersson, Jesper Nygård, Magnus T. Borgström, Heiner Linke, Anders Mikkelsen

**Affiliations:** † NanoLund, Lund University, Box 118, 22100 Lund, Sweden; ‡ Division of Synchrotron Radiation Research, Department of Physics, Lund University, Box 118, 22100 Lund, Sweden; § Center for Quantum Devices and Nano-science, Niels Bohr Institute, University of Copenhagen, 2100 Copenhagen, Denmark; ∥ Division of Solid State Physics, Department of Physics, Lund University, Box 118, 22100 Lund, Sweden; ⊥ School of Information Technology, Halmstad University, Box 823, 301 18 Halmstad, Sweden

**Keywords:** optoelectronics, nanowire, communication, III−V, neuromorphic, nanophotonics, FDTD

## Abstract

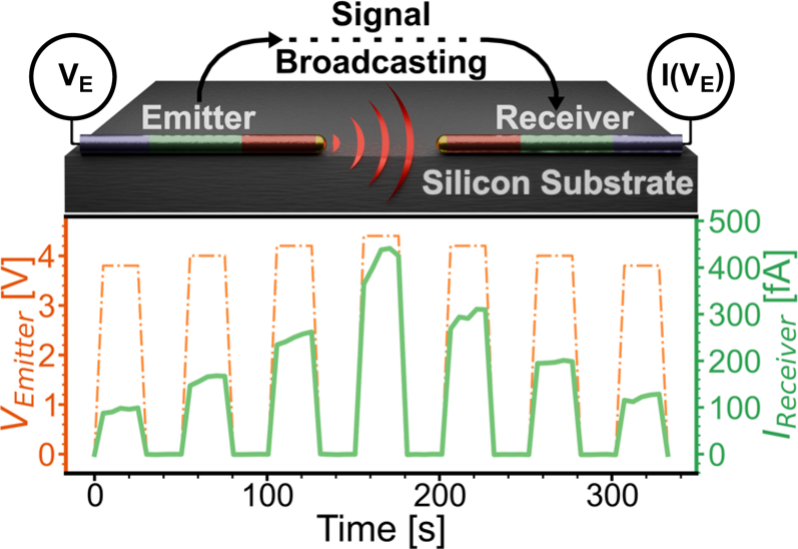

On-chip optical communication between individual nano optoelectronic components is important to reduce the footprint and improve energy efficiency of photonic neuromorphic solutions. Although nanoscale photon emitters and receivers have been reported separately, communication between them remains largely unexplored. We demonstrate direct on-chip directional broadcasting of light between individual InP nanowire photodiodes on silicon. The performance of multiple wire-to-wire communication circuits is mapped, demonstrating robust performance with up to 5 bit resolution as needed in biological networks and a minimum component driving power for continuous operation of 0.5 μW which is below that of conventional hardware. The results agree well with theoretical modeling that allows us to understand network performance limits and identify where significant improvements could be achieved. We estimate that an energy per operation of ∼1 fJ and signal fan-out from one emitter to hundreds of other nodes is possible. We find that the nanowire circuit performance parameters can satisfy the quantitative requirements to run the tasks of neural nodes in a bioderived neural network for autonomous navigation.

## Introduction

On-chip nanophotonic solutions can potentially improve speed and energy efficiency of computing far beyond what is possible with present day CMOS, especially for realizing artificial neural networks (ANNs).^1−4^ While transistors have high switching efficiencies, electrical communication between them (and with associated memory cells) is much more energy consuming and scales nonlinearly.^1,5^ Because neural networks are based on large interconnectivity, this can result in high energy consumption.^5,6^ Further, electron-based neuromorphic systems tend to be slower than what is possible using photonics,^2^ again due to the communication between components.

Using Si waveguide circuits and macroscopic optical elements, significant progress has been demonstrated for running photonic ANNs.^2,4,7,8^ However, already networks with four input/outputs are millimeter scale.^4^ Concepts of using overlapping light signals between nanoscale neural nodes inside a single waveguide^9^ (a broadcasting scheme) could shrink the network size several orders of magnitude, as could the use of light multiplexing.^1,2^

Because the transmission of light is readily done with low losses, a key challenge for realizing energy efficient optical computing is the conversion between electrons and photons that occurs in the emitters/receivers for the evaluation, emission, and amplification of signals.^1^ In particular, the photon emitters are macroscopic, often external, components that fundamentally will draw significant energy^10^ due to their size. Distributing signals on the chip from such emitters is possible, but leads to large footprints due to waveguiding lines. Additional components for manipulating or amplifying light signals in neuromorphic solutions are often hundreds of microns in size.^1,5,11−13^ To resolve this, implementing subwavelength (nanoscale) optoelectronics has been deemed necessary arguing that their smaller size can reduce both footprint and power consumption orders of magnitude.^1,3,4,13^

The fundamental starting point for the development of on-chip optical communication between multiple nanoscale (subwavelength) components is the optical communication between an emitter–receiver pair. In addition to being a central technological step, this also helps set experimental limits for the efficiency and viability of intercomponent communication. From this information, realistic models of larger neural networks can be created and used to estimate energy efficiency, feasibility, fabrication, and design limitations.^9^ To evaluate the potential of the components in a network, we must (a) understand their individual optoelectronic function, (b) demonstrate analog signal transmission with a range of signals in the 4–5 bit range needed for biological neural networks,^14^ and (c) use the experimentally observed efficiencies to estimate the energy efficiency and fan-out of an optimized system.

Surprisingly, such on-chip optical communication between nanocomponents has not yet been experimentally realized, although it was proposed years ago^15,16^ and the benefits of going to the nanoscale are acknowledged.^4,13^ For larger hundred micrometer-scale components, on-chip light emission and reception was also an important step which has been achieved.^17^ Creating nanocomponent communication circuits includes several significant challenges: A high yield of functioning nanoscale light emitters and receivers is needed to have a complete circuit with many simultaneously functioning components. Rapid/flexible/precise nanostructure positioning and high-quality electrical connections to the exterior world are needed in the circuit design.

For use in optical neural networks, direct bandgap III–V compound semiconductors are particularly suitable due to the very high electron-photon conversion efficiencies possible. III–V nanowires are presently one of the most mature nanodevice technology platforms beyond silicon CMOS^18^ with high suitability for nanophotonic applications and direct integration into silicon.^19−21^ Nanowires have shown advantages both in terms of efficiency, wavelength variability, directionality and polarization sensitivity^22,200^ of both receivers and emitters.^18,23−26^ Importantly, InP nanowires have been demonstrated as high performance photodetectors (photovoltaics),^27^ but can also simultaneously work as light-emitting diodes (LEDs).^28^ LEDs are favorable as emitters in neuromorphic systems when confined to the nanoscale due to their low energy consumption and fast response.^10^ Modeling (using experimentally benchmarked device properties) suggest that III–V nanowire optoelectronics can implement a full neural node receiving, emitting and evaluating optical signals with energy efficiency and speed far beyond present hardware.^9^ The node was simulated in an artificial neural network derived from the navigation capabilities of the insect brain. This central complex (CX) neural network is found across insect species, and it allows the insect to return back after foraging with limited and noisy optical information. It was found that shrinking emitters/receivers to the nanowire size and using the excellent conversion properties of InP, energy consumption would be far better than with standard hardware and on par or even better than biological brains.^9^

As a guiding point, the highly energy-efficient biological systems, such as the human brain, has an energy use per synapse operation of ∼30 fJ and ∼60 pJ for generating a neuron spike.^5,29,30^ Exact comparison to energy consumption of artificial hardware (including optical) is complicated, but the biological brain is generally found to be orders of magnitude more efficient.^1,31−33^ Using nanoscale optical emitters/receivers energy consumption on par or better than biological systems is within reach for optical solutions as the energy consumption in light emission/reception can be minimized.^1,2,9,13^

In this article, we present complete emitter-receiver circuits consisting of InP p-i-n photodiode nanowires, originally developed for high efficiency photovoltaics.^23,26,34,35^ The nanowires are placed in specific positions on a Si substrate with a 1000 nm thick insulating thermal oxide. The emitters are aligned to optimize the transmission as their specific geometry promotes light emission along their long axis. Consistently good electrical contacts to both the n- and p-type segments are achieved for electrically driving the components. First, the electrical behavior of the individual nanowires and their ability to emit and receive light is characterized. We then demonstrate how light signals (of different intensities) can be transmitted between the nanowires separated by distances up to 2100 nm and in both parallel and perpendicular receiver configurations. Mapping out the properties of nine communication circuits and for prolonged operation, we observe high reliability. Using finite difference time domain (FDTD) simulations, we estimate the light transmission between the nanowires exploring dependence of nanowire placement as well as the effect of their metal electrical contacts. The modeling in conjunction with estimates for emission/absorption efficiency indicate that further improvements by several orders of magnitude is realistic by confining the light in the chip plane and improving emitter efficiency. The values derived for the circuit performance show that it can run a biologically inspired network of the insect brain CX used for navigation.

## Results and Discussion

### Circuit Fundamentals and the Basic Nanowire Device Used

**Figure 1:**
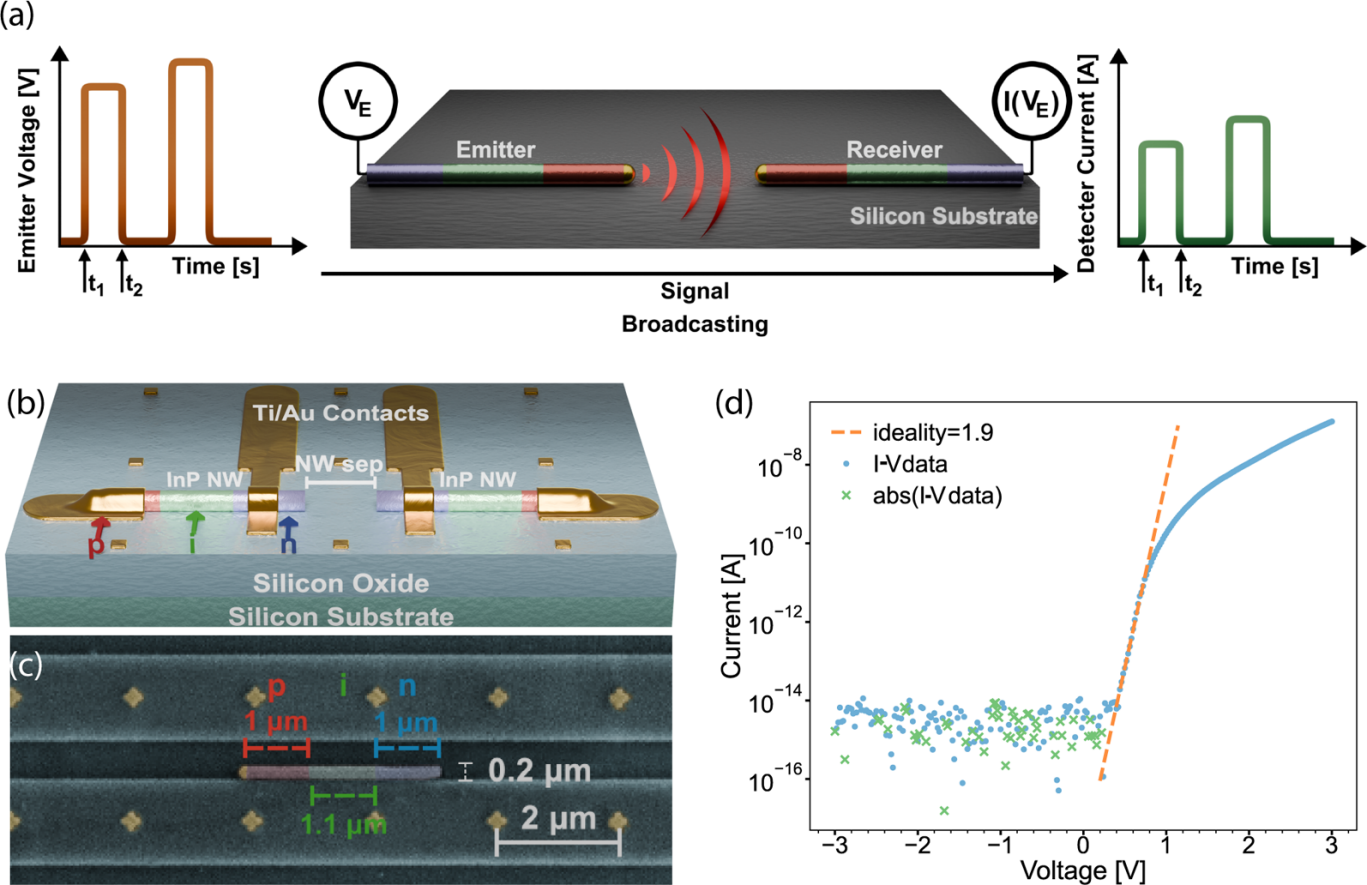
Fundamental circuit schematics, and InP nanowire photodiodes used for the communication: (a) Illustration of optical broadcasting and communication between two subwavelength (nanoscale) components. Initial proof of concept for on-chip nanostructure-to-nanostructure light communication, from which the function of larger networks can be inferred. Controlling the emission light pattern by the geometry of the emitter, can be used to direct the light, illustrated here by the use of nanowires (b) Detailed render of the proposed circuit, realized as a nanowire-to-nanowire composite device. Separation between NWs varies between devices, the largest distance being 2100 nm. (c) False color SEM image of a single InP nanowire photodiode, whose p-i-n segments are indicated, aligned in an oxide trench. (d) Electrical measurements of an individual nanowire after contact metallization. The low bias regime was used to fit the ideality factor, as seen in the graph.

The basic concept in this paper of light signaling between nanonodes is illustrated in Figure 1a. In the present case, nanowires were designed to emit in a dipole pattern from their ends. Shaping the nanowire geometry can be used to tailor the light emission pattern for specific network communication directions.^36^ When a voltage is applied to an emitter, light is broadcast, which then excites the current signal in a receiver. The efficiency of the photon-electron conversion (along with losses in the light propagation) is central to the energy efficiency of an optical network, which should also allow 4–5 bit analog communication. The devices should be placed on a SiO_2_/Si substrate preferably using fabrication techniques that allow them to be integrated on top of a CMOS chip in the future.

In the present study, we use InP nanowire p-i-n photodiodes that work as efficient photodetectors,^37,38^ but can also work as light emitters.^34^ The nanowires were grown from Au seeds on a separate substrate, as described in the method section. A detailed sketch of the communication circuit realization is shown in Figure 1b, including both nanowires and electrical interconnects. The distance between the wires can be varied, but for evaluating the circuit function, they were positioned with a separation of about 1 μm. Nanowires were mechanically removed from the growth substrate using the tungsten probe of a micromanipulator and placed into prefabricated oxide trenches on the Si wafer surface for alignment. In Figure 1c, a scanning electron microscopy (SEM) image shows a positioned nanowire photodiode in an oxide trench on the substrate. From such SEM pictures, the precise orientation and geometry of the components can be mapped out, electron beam lithography (EBL) mask patterns are created on top, and Ti/Au contacts added in a final fabrication step. The procedure is described in more detail in Methods. I–V measurements of the InP nanowires verified their diode behavior, as exemplified in Figure 1d. I–V diode behavior was observed for all circuits with functioning communication, but quantitative parameters such as the ideality factor varied. The ideality factor was quantified for a number of devices (see Table S1) and found to be in the range of 1.5–3.6. This is in good agreement with other studies on this type of nanowires.^34,39^ It indicates that variations in the basic diode parameters still allows communication. Variations are likely due to contact and surface conditions, not the wires themselves.^34,39^

### InP Nanowire Device Function as Light Emitter and Receiver

**Figure 2:**
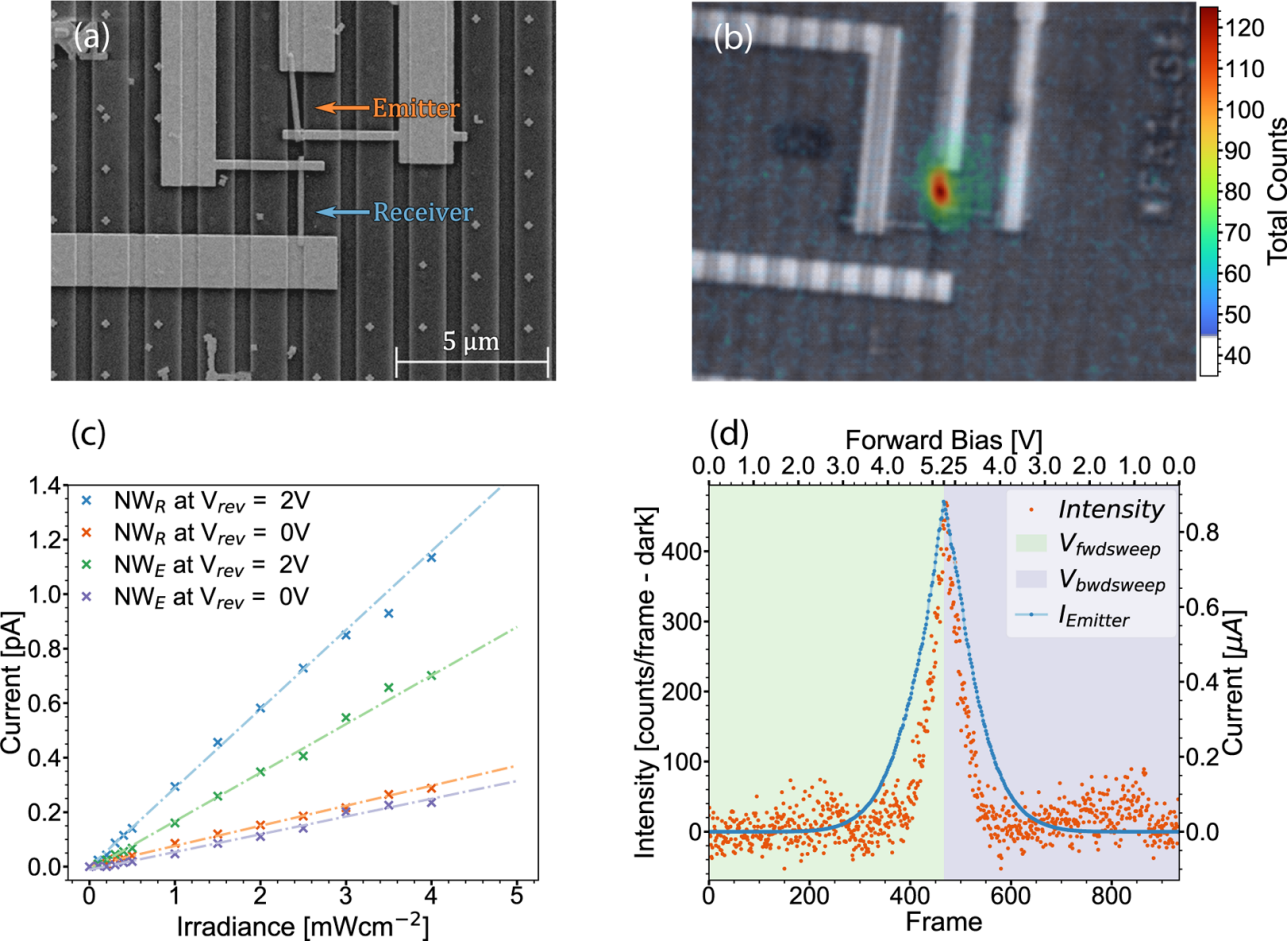
Evaluation of emitter and receiver nanowires of the complete communication circuit. (a) SEM image of a two-nanowire device circuit, whose emitter and receiver are labeled. In this case the wires are separated by 550 nm, but separations of up to 2100 nm has been investigated and show communication. (b) External light emission from the emitter nanowire, represented as the sum of counts over the dark noise detected by a high-resolution optical microscope. Emission is superimposed over a brightfield image of the same circuit. (c) Photocurrent at 0 and 2 V reverse bias in the receiver nanowire as a funtion of irradiance of an external light source, with linear fits for each measurement series. (d) Emission light intensity detected by the optical microscope setup, as a function of the applied voltage. The I–V curve was captured simultaneously during light detection.

Figure 2a shows an SEM image of a fabricated circuit, as described in Figure 1b. The distance and alignment between the wires can be controlled with good precision They can consistently be placed within 1 μm of each other, rotational alignment vary by ±5°. The 180° orientation of each wire could not be controlled and was instead verified using SEM afterward. The distance and orientation between the devices are chosen to achieve a high degree of light transmission, resulting in the largest possible output currents for detailed signal evaluation and ensuring that nanowires with lower performance can still be studied.

A specialized optical microscope setup (see methods) was used to verify the light emission properties of the nanowires. As seen in Figure 2b, light emission from individual nanowires was observed externally. With the Au contact geometry obscuring the ends of the NW, the observed out-of-plane emission near the intrinsic region is as expected. The emission as a function of nanowire I–V was also measured in this setup. Figure 2d shows that the light emission is proportional to the current across the diode. The absolute light emission from one wire to the other is difficult to determine experimentally, as we only image the far field out-of-plane emitted light. A calibrated solar simulator (see Methods) was used to verify the operation of the nanowires as receivers. We measured the light-induced current for increasing irradiance at zero and reverse bias, as shown in Figure 2c. The measured behavior corresponds to the expected nanowire photodiode performance and thus confirms the fundamental optical properties as emitters and receivers. We also use these characteristics to estimate external quantum efficiencies (EQE) of the nanowires, as discussed below.

### Nanowire-to-Nanowire Communication Demonstrated

**Figure 3:**
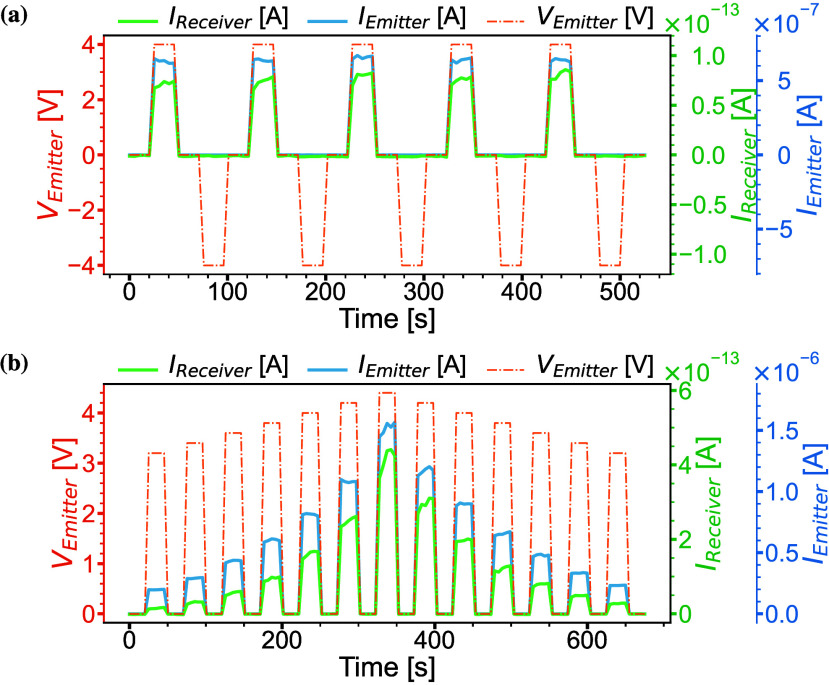
Time-dependent emitter and receiver measurements in a communicating two-nanowire device system. (a) Measurement where emitter nanowire is pulsed in the range [−4, 0, 4] V, showing the resultant current measurement in the receiver nanowire. (b) Receiver and emitter current measurement when emitter voltage is stepped from 3.2 to 4.4 V, starting at the detection threshold in the receiver. Receiver nanowire is reverse biased at 2 V.

In Figure 3, we show the communication between individual InP nanowire diodes in the configuration shown in Figure 2a. We demonstrate optical communication using a voltage protocol on the emitter nanowires that switches between 0, +4 and −4 V (see Figure 3a). The receiver nanowire is reverse biased at 2 V. No emission of light occurs when operating the emitter at zero or reverse bias, as established in the previous section; see Figure 2c. When running the emitter nanowire at a forward bias, a current is observed on the receiver nanowire (green curve in Figure 3a). The communication response is consistent and stable when tested for more than a day without any significant performance drop (see Figure S11 in the SI). In Figure S2 and Table S1 of the SI we show results from 9 different circuits demonstrating communication. Clear signal transmission between wires separated up to 2100 nm and aligned both parallel and perpendicular to each other was also observed (see the devices in Figure S9). The main limitation in the number of functioning circuits produced was fabrication reliability and failure of one of the many steps in the process. If all steps worked, high proportion of devices on a chip would function.

Differences in response of different devices can be attributed to variations in the position and orientation of the nanowires, in addition to the varying electrical performance of the individual InP nanowire devices. While single devices show fluctuations in performance due to contact and surface variation, getting repeatable operation from device to device is possible,^34,40^ but would require further fabrication optimization. Some current noise is also observed in both the emitter and receiver currents during operation, as seen in Figure 3. The noise floor is estimated to be ±5 fA. This can be attributed to the wires not being encapsulated and thus, exposed to the ambient air. Additionally, a small increase in the receiver current is observed during the time the emitter is biased. This can be related to small changes on the surface of the nanowires as current passes through them and light excites them, in agreement with previous work.^41,42^ This could potentially be resolved by adding additional layers for surface passivation.^43^ Finally, no current response was observed on the receiver nanowire when the emitter was reverse biased, indicating that there is no electrical cross-talk between the wires. This was further investigated by direct measurements between the nanowire contacts and the silicon substrate underneath the oxide. For these measurements, leakage currents were insignificant, as no current response was observed.

In Figure 3b, we demonstrate transmission of signals of different strengths. The emitter is biased with pulses of increasing voltages from 3.2 to 4.4 V, in steps of 0.2 V. This shows that the present setup has a suitably analog range for communication. From the smallest steps discernible in this experiment (10 fA), which is consistent with the noise floor and the range of current of 400 fA, we estimate that signals can be transmitted with at least 40 (∼5 bit) uniquely distinguishable levels based on the present circuit (Figure 3b). This is similar or higher than the range of distinguishable values in a synapse of the human brain of 4–5 bits.^14^ To further investigate this relationship, we have also linearly swept the voltage of the emitter and measured the detector current at different voltages (Figure S5(b) in the SI). This can be compared to the measurements of the receivers I–V as a function of the intensity of an external light source at similar wavelengths. First, we can observe that the I–V response of the receiver nanowires is similar, regardless if the light source is external or an on-chip nanowire emitter. The response of the receiver is smooth and predictable with a significant range of discernible intensities. This again confirms that the receiver observes a light signal and that the nanowire emitter behaves much like a standard diode. The conversion between emitter and receiver current is not completely linear, and at high light intensities an increase in receiver current over time is also observed. This can be attributed to surface trap states, as commonly observed for nanowire structures.^44^

### Efficiency and Modeling of Signal Transmission

The relative difference of the injected current in the nanowire emitter to the current of the nanowire receiver gives a simple experimental measure of the signal transmission ratio of ∼10^–7^, providing an estimate of the nanowire-to-nanowire signal coupling ratio when using light. This is a starting point for estimating the coupling efficiency of the circuit and identify where significant losses occur. To understand and estimate the efficiency, we must consider light transmission efficiency between components as well as the EQE of photon-electron conversion in each components.

We started by delving into the light transmission process between the nanowires and associated losses. First, it must be noted that some of these ”losses” are by design, especially those attributed to in-plane light emission. We envision a broadcasting strategy in which a single emitter shares its light emission with several receivers in a neural network. Thus, only a small percentage of light from an emitter is available to an individual receiving node. The exact fraction available depends on the network design, however, as an example the insect brain can have up to 200 connections per neuron.^45^ In the CX navigation circuit of the insect brain, each neural node shares its light emission with up to 11 other nodes.^9^ Here we note that the dipole-like emission of the present nanowires is well suited to achieve the weighted light distribution of such a navigation network.

The absence of any light confinement mechanisms in the plane of the chip substrate leads to significant out-of-plane optical losses. In a 3D network, this light could be used for connectivity by stacking devices, but for a 2D configuration on a chip, the light must be confined in the plane. This can be done by embedding the devices in a blanket transparent oxide waveguiding material (e.g., Al_2_O_3_ or SiO_2_) which strongly limits the out-of-plane losses.^9^ For the current study, the free-space emission without any 2D waveguiding was useful, as it enables easy external monitoring of the nanowire light emission.

**Figure 4:**
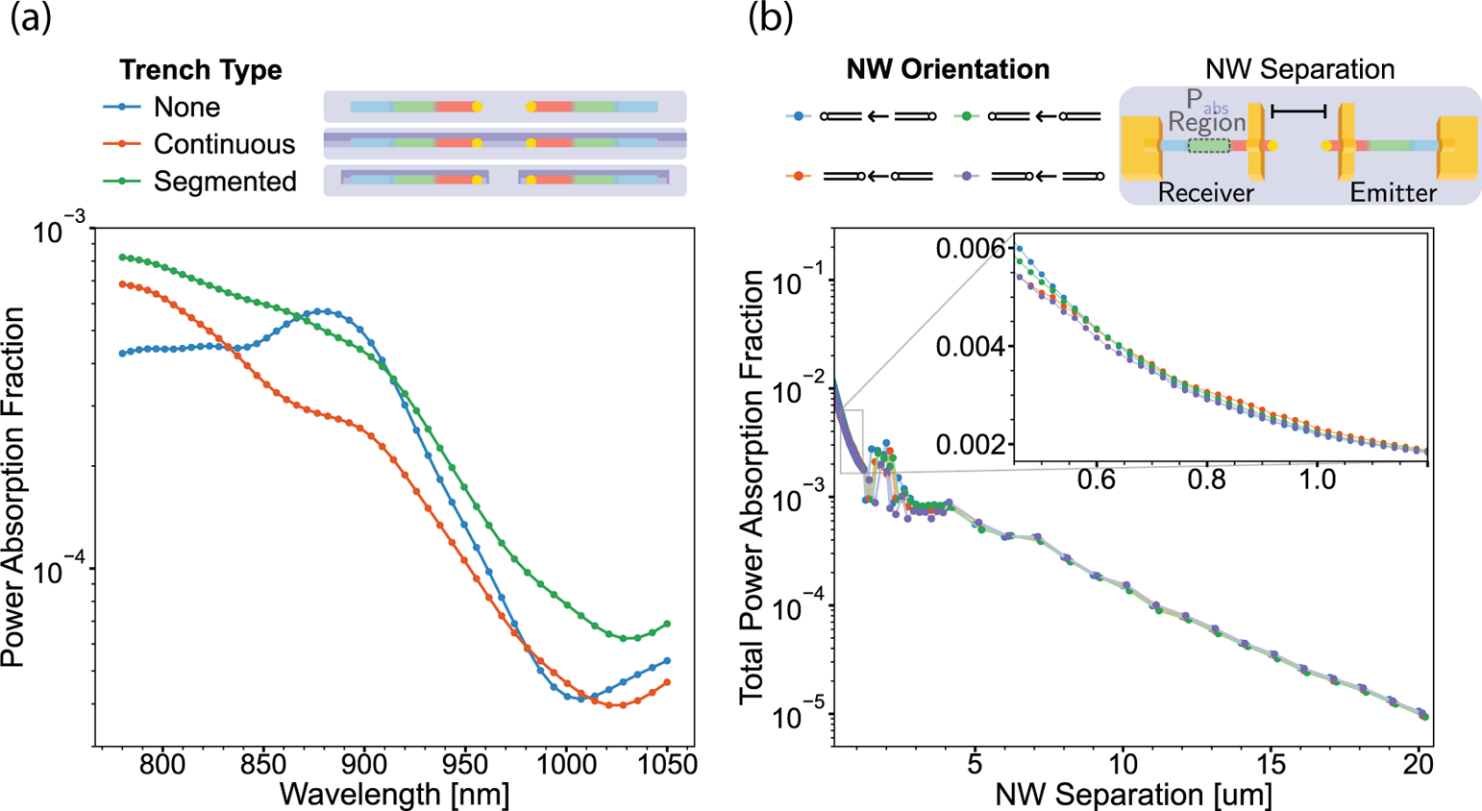
FDTD simulations of a nanowire (NW) emitter-receiver pair calculating the fraction of the power of emitted light that is absorbed by the receiver (*P*_abs_). (a) *P*_abs_(λ) for devices realized on different substrate surface geometries, where nanowire separation is 800 nm. As illustrated in the legend, “None” models the device on a planar and featureless SiO_2_. “Continuous” models nanowires in a 100 nm deep and 600 nm wide trench, akin to fabricated devices. “Segmented” models nanowire in small rectangular trenches of the same width and depth as before, but with a length of 3.5 μm, and separated by 800 nm of oxide. (b) Total *P*_abs_ in the receiver nanowire evaluated at different separations and relative orientations of emitter and receiver nanowires; integrated over wavelength range 780–1050 nm. Inset shows a zoom-in on the region of the present devices. The schematic in the legend illustrates 165 nm thick gold contacts that approximate the device contacts on fabricated devices, along with nanowire separation.

FDTD simulations of the fabricated devices were used to evaluate the fraction of transmitted light and, consequently, the scope of using these components in larger photonic circuits (details on the simulations are found in the methods). In Figure 4a, we show the wavelength dependent efficiency (power absorption fraction, *P*_abs_) as a function of wavelength, using a simulation geometry consisting of two nanowires, separated in space by 800 nm, under different substrate configurations, including those shown in Figure 2a. A high power absorption fraction is seen at wavelengths of 950 nm and below for all configurations (consistent with the bandgap of InP). The absorption from the wires is strongest in the range of 850–1000 nm, with a maximum at 930 nm,^46^ which is well captured. The observed differences between different substrate configurations indicate how the power absorption can be altered by the geometry of the surrounding oxides.

The light transmission dependence on the emitter-receiver separation, as well as nanowire orientation with the electrical contacts included, was also studied, as shown in Figure 4b. Similar calculations were carried out without contacts, as can be seen in Figure S3 in the SI. We find that the contacts have a limited effect on overall light transmission, as long as they do not cover the ends of the nanowire. However, the presence of gold contacts in the simulation does focus the signal in the forward direction. This can be explained in that the contacts act as light reflectors, focusing and reflecting outward bound light toward the receiver. The Au seed particle at the end of the wire also influences the light transmission in particular if no contacts have been added. The receiver seed induces forward scattering of incoming light, focusing light onto the nanowire. In the emitter, the seed acts as a reflector, redirecting zero axis outward radiation back toward the device center. This all illustrates a potential mechanism for creating specific geometric communication patterns, using plasmonic metal nanostructure antennae effects.

In summary, a coupling efficiency in the light transmission between two nanowires of about 0.2% for the present devices is found. Additional simulations were performed (see Figure S10 in the SI) to establish the potential improvements of the coupling efficiency by adding a blanket oxide film overcomplete devices via e.g. atomic layer deposition (ALD).^47^ The oxide film can act as a waveguide to confine the light increasing and it is indeed seen that an increase of a factor of 20 in coupling efficiency can be achieved in this way for the devices.

While the simulations give a robust estimate of light transmission efficiency, further considerations are needed to estimate the coupling of the final electrical currents in the emitter and the receiver. So, we must incorporate the electron–photon–electron conversion losses that happen in the nanowires. In a simple consideration, the overall transmission strength (*T*) is
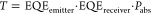
1

From the current response of the receiver due to an external light source, we estimate the EQE of the receiver to be between 14% and 20% for a central wavelength of 910 nm (see Section 3 in the SI for details). Using eq 1, we can then get an estimate of the EQE of the emitter to be between 0.019–0.035%, using the receiver results and the estimated fraction of light broadcasted between the wires (Figure 4). Thus, for the present devices, the main losses are in the light escaping out-of-plane and the electron-photon conversion of the emitter nanowires.

The observed receiver efficiencies are also lower than the EQE values that can be achieved for large arrays of upright standing InP nanowire arrays (60–80%), however they are a very good result as the EQE is known to depend strongly on the surface termination of the wires as well as their contacts.^43,48^ For the emission much higher values (40%), have been observed for nanowire structures tailored for emission^18,49^ as compared to what we find in the present case. This is not surprising as the present type of wires have not been optimized as emitters.

To estimate power use, from Figure 3b we can see that the lowest emitter current that transmits a detectable signal to the receiver is 0.1–0.3 μA at ∼3 V, which result in a power consumption of ∼0.5 μW under continuous operation. In a spiking configuration, this power consumption would be reduced substantially as the device needs only provide light to generate the spikes and not continuously. To assess power consumption in a spiking-based system using the present components, we estimate the energy use per operation (defined as spike generation, transmission, and evaluation). We further base an estimate on the observation that the incoming light signal needs to perform a nonlinear operation of turning a transistor on to generate an output signal. In previous work, an artificial neuron that performs such a task using nanowires (as in the present case) has been proposed and simulated.^50^ The field effect transistor (FET) wire used for the nonlinear signal evaluation in that case has a capacitance of <100 aF from which it can be derived that ∼600 electrons are required to open the transistor and give an output signal. With efficiencies of the components as found above, this results in a total energy usage of ∼60 pJ per operation. Returning to the estimated power consumption of ∼0.5 μW, this would correspond to an on-time of ∼1 μs for a spike, which is well within the response time of a InP nanowire (which is <1 ns).^50^

Another relevant parameter is the maximum distance that the individual nanowires can communicate without any intermediate amplification. For the present device performance and geometry, assuming that the smallest signal that can be measured is ±5 fA, emitting a signal of 2 μA at 4.4 V, we get a distance of 12 μm (see SI, Figure S8). Based on this, we also estimate the number of nodes with which the present nanowire could communicate in an open broadcasting structure. Using that the nanowires have dipole like emission from their ends, one can estimate the area in which receivers can detect a signal. Assuming then that each receiver node takes up a 3 × 3 μm square and it absorbs only a small fraction of the light (as seen in Figure 4) where each emitter can directly communicate with 12 nodes.

While the values described above represent the performance of the present nanowire circuit, the known ultimate possible performance for individual components and the transmission (as discussed above) indicate that significant improvements are possible. Adding a quasi 2D waveguide to confine the light in the plane of the chip can increase transmission by a factor of 20 in previously simulated device configurations (see Figure S10 in the SI). Improving surface passivation, wire design, and contacts can significantly improve EQE values for both emitter and receiver, enhancing the receiver EQE by a factor of about 3 and the emitter EQE by a factor of 2000. Combining these factors, a total improvement in the transmission efficiency of 120000 can potentially be achieved. Allowing for some uncertainty in the possible optimization of each of the three parts, an improvement of 10000 seems more probable. Using the experimentally verified possible values for transmission and electron-photon conversion, an energy use of ∼1 fJ per operation would then be realistically achievable. Using the estimate for how absorption fraction depends on distance (Figure 4b) an increase in transmission strength by a factor of 10000 increases the area reachable by the dipole emission of an emitter by a factor of 25, allowing communication with up to 300 receivers without using additional amplification or lateral waveguiding. There are more than 200 connections found in insect neural networks. Finally, we confirm that the performance possible to achieve using the present components should be high enough to run a previously proposed neural network mimicking the central complex of the insect brain is well within reach.^9^ In this concept, maximum distances between devices are around 6 μm, while the necessary current induced in the receiver should be 20 pA in a continuous operational scenario. While the distance is already in range, 1 order of magnitude improved transmission is needed to realize the necessary current. This should be realizable with known fabrication improvement, such as embedding the circuit in a quasi 2D waveguide.^9^

## Conclusions

In summary, we demonstrate optical communication between individual nanowire photodiodes on silicon substrates. In the present work, both the transmission efficiency and the electron–photon conversion efficiencies of the components are below the known optimal values. Thus, further work could realistically improve efficiency by a factor of 10000. While the InP nanowires used, have very high reliability and can perform as both emitters/receivers due to years of development,^18^ other nanostructure components will be reaching a similar level of maturity making them candidates also for network circuits. From the present work, we can also estimate the prospects for scaling up to larger circuits. While our measurements show that networks of tens or hundreds of components is within reach with realistic performance improvements, this would demand a high yield in functioning devices. Because the nanowires are homogeneous, if all fabrication steps are successful, most components will function well, thus allowing for larger networks. It is noteworthy that for implementation of biologically inspired networks, such as from the insect brain and using the concept of light broadcasting between assemblies of nanophotonic components,^9,50^ a significant variation in both placement and function of each wire can be tolerated. Using a broadcasting concept allows a significant shrinking of the footprint of optical computing systems, one of the primary challenges of integrated photonic circuits. Our communicating wires can be spaced with distances of less than 6 μm for the navigation circuit and a total functioning navigation circuit would be less than 100 μm in diameter.^9^ In terms of energy use, our new circuit is already competitive with present technologies, which have uses in the nJ to pJ range per operation. Further realistic optimization would decrease energy use by orders of magnitude. In particular, a focus must be on the electron-photon conversion as that is their main obstacle for energy efficient optical networks.

While the present work was done using InP nanowires on Si substrates, the results should be generally applicable and scaleable. In general, several strategies for combining InP and other III–V nanowires with Si and in particular the CMOS platform have been explored.^8−12,51,52^ Either III–V materials can be grown on top of Si substrates with CMOS circuitry beneath^9−12^ or the III–V nanowires can be grown on separate substrates and then subsequently transferred to a CMOS substrate.^5,8^ Large-scale assembly of electronic circuits using various types of wires and tubes has been reported, also in conjunction with CMOS.^4−8^ A further relevant point is the preparation of the substrate and preparation of relevant oxides, both for electrical isolation as well as for waveguiding (as discussed above). Various low temperature (<300 °C) oxide synthesis techniques are used in industry (such as ALD). As fabrication of III–V circuits as discussed here are compatible with Si CMOS this allows optical communication in combination with highly advanced electronic circuits. As a result, the work opens up for scientific exploration of larger intercommunicating optical circuits using nanoscale optoelectronics. While emphasizing that the component reliability and the ability to place and contact them electrically are important, the efficiency of the components can be low while still yielding a functioning network.

## Methods

### Nanowire Growth

InP nanowire arrays were grown by using metal–organic vapor phase epitaxy (MOVPE). A hexagonal pattern of Au nanoparticles with a pitch of 500 nm was defined on a 2” InP (111)B substrate using displacement Talbot lithography and E-beam evaporation of Au.^27^ The nanowire arrays were grown in a low-pressure (100 mbar) Aixtron 200/4 MOVPE reactor, using the III–V precursors trimethylindium (TMIn, χ = 5.94 × 10^–3^) and phosphine (PH3, χ = 6.92 × 10^–3^). Tetraethyltin (TESn) and diethylzinc (DEZn) were used to dope the nanowires. The precursor gases were transported using H_2_ carrier gas with a combined flow rate of 13 l/min. The Au particle positions were preserved with a prenucleation step at 280 °C, followed by annealing at 550.^35^ Nanowire growth then commenced at 440 °C. Nanowires with a diameter of 200 nm and length of 3000 nm were doped in a p-i-n structure with segment lengths of 1000 nm (p-type), 1000 nm (intrinsic), and 1000 nm (n-type), respectively. In situ monitoring using a LayTec EpiR DA UV optical reflectometry system was used to control the length of each segment to the desired length [Heurlin 2015]. The molar fractions of the dopants were χ_DEZn_ = 1.1 × 10^–5^ (p-type), χ_DEZn_ = 0.3 × 10^–7^ (intrinsic), and χ_TESn_ = 4.3 × 10^–5^ (n-type), respectively. The Zn doping in the intrinsic segment acts as compensation doping of unintentional p-vacancies, resulting in a slight n-type doping of nonintentionally grown segments.^35^ HCl was used at a molar fraction of χ = 1.23 × 10^–4^ to suppress radial growth.^46^

### Device Fabrication

Nanowire communication circuit fabrication was done on 5 × 5 mm Si chips featuring four distinct flower-like device regions, each with 20 petal-like large-scale contact leads intended for wire bonding. These pads connect to a central 100 × 100 μm writefield region, within which the nanowire device circuits were defined.

The 5 × 5 mm^2^ chips were diced from a larger 3″ silicon wafer prepatterned with two separate lithography steps. First, an optical lithography step performed with a maskless aligner (MLA) exposed a GDS mask on a bilayer of LOR10b and S1813. This established the large scale 5 × 5 mm^2^ grids containing a configuration of 2 × 2 flower patterns over the entire wafer. Following this were metallization and lift-off of Ti/Au (10/200 nm). The subsequent electron beam lithography (EBL) step, done on a bilayer of PMMA 200k and PMMA 950k, defined the alignment markers around the central writefield region of each flower pattern on the whole wafer. This allowed for the evaporation and lift-off of Ti/Au (10/40 nm) before the wafer was diced.

On a diced chip, 200–400 nm wide trenches were patterned via EBL onto ARP-6200. 100 nm deep trenches were etched into the oxide using reactive ion etching (RIE). These structures act as alignment guides for the InP nanowires, placed manually in the trenches using a micromanipulator equipped with a fine-tungsten tip. A final EBL step was used to pattern the nanowires for electrical contacts of Ti/Au (5/160 nm). A detailed analysis of additional fabrication specifics will be presented elsewhere. For all present devices, Si wafers with 1000 nm of thermal oxide was used to ensure that there is no current leakage through to the substrate, even when using etched trenches for nanowire alignment.

### Device Measurements

The electrical setup used for the optical communication measurements consists of a Cascade 11000B probe station outfitted with four DCP-100 probes. Each probe is connected to a separate source measure unit (SMU) component in a Keithley 4200-SCS semiconductor characterization system, allowing for simultaneous biasing and measurements on four channels. The circuit diagram for this electrical characterization system is presented in the SI in Figure S1. The thick oxide layer on the chip isolates the devices from the underlying substrate material. This consequently means that electrical measurements on the devices can be considered to have a floating ground when the stage contact is set to the system ground. Before electrical characterization, each device was tested for leakage currents by connecting the stage contact to the common ground of an SMU and measuring the current response with a biased device contact. For all characterized devices, these measurements do not show current values above the noise floor. Standard measurement protocol assigned both finger-contacts nearest to the center of the device to the common ground, as illustrated in Figure S1, whereas biasing and current measurements were done on the contacts away from the center.

### Standardised Nanowire Testing

All devices were subject to a standardized protocol developed for consistency across devices. Each nanowire of a device pair was first tested for diodic behavior. To avoid damaging nanowires during these tests, the current compliance was set to 1.1 μA on the Keithley. A high bias voltage sweep was then initiated over each connected nanowire, usually in the range of [−7 , 7] V, to bridge any oxide in the contacts of the nanowires. The subsequent I–V measurements would be characteristically diodic if a good metal–semiconductor interface was formed during metallization. Consequently, when remeasured I–V curves were reproducible without major hysteresis, a maximum allowable voltage *V*_max_ was determined, for a current value of 0.8 μA, for subsequent measurements. Next, we measured leakage current by biasing only the outer nanowire contact in the operation range to verify that the measured current on the ground contact was not above the noise floor. Once a device with two functioning diode nanowires was identified, reverse bias sweeps were performed in both dark and illuminated conditions on both components. This was used to identify the nanowire better suited to operate as a receiver based on the difference and magnitude of the induced photocurrent under these conditions.

Following this, all four terminals were connected according to Figure S1 for the pulse test. The emitter nanowire was biased with a sequence [0, *V*_max_, 0, −*V*_max_], while the receiver nanowire’s outside contact had a fixed applied reverse bias of 2 V. Both emitter and receiver channels were then set to record currents as the emitter voltage sequence was repeated five times. For all measurements, the Keithley was set to quiet acquisition mode, with both the hold and sweep times set to 2.5 s. Once communication was confirmed, a “ladder measurement” was done, with the emitter biased under 8 distinct voltage steps, from the receiver detection threshold to the emitter current compliance. Finally, the receiver response was recorded with the emitter under a continuously increasing forward bias, the results of which are shown in the SI. Here, the emitter was swept in forward bias with a step size of 0.025 V in the range [0, *V*_max_]. The reverse bias on the receiver was also stepped by 1 V in the range of [0, −4] V.

To approximate the flux density that reaches the receiver from the emitter, the photoresponse of the nanowires at known power densities was tested by using a two-probe G2 V Pico variable solar simulator. The two probes were connected to a Keysight B2980A SMU, and measurements were controlled by python code. From this, photocurrent measurements at specific reverse bias and irradiance was correlated. In addition to testing the leakage current, the light emission from the nanowire was also verified. This was done by mounting the sample on an STM holder attached to a UNISOKU measurement system, wherein the emitter nanowire was biased under a microscope with a ×100 objective and attached to a standard CMOS camera.^53^

### FDTD Simulation Parameters

Three dimensional simulations were done in the commercially available Lumerical FDTD Solutions, which solve time-dependent partial differential Maxwell equations in order to estimate light interactions over a range of wavelengths in given nanoscale structures. Consequently, these simulations allow for accurate estimates of light transmission and absorption through various device architectures. The simulation geometry was designed to replicate real device dimensions as closely as possible. To achieve rapid prototyping and fast replication of experimental conditions, all simulation geometry was defined programatically from a list of parameters such as nanowire radius and length, trench depth and width, and oxide thickness. Nanowires were modeled as cylinders with a spherical seed particle of the same radius placed at the center of the end face of the cylinder. The dimensions of these wires were assigned to match the nanowires we utilized in our device fabrication. Since simulations dealt with various substrate geometries as well, such as 100 nm deep oxide trench structures, wire placement was always done such that the base was in direct contact with the substrate oxide. Material constants for the different InP, Au, SiO_2_, and Si device components were taken from Palik.^54^ Gold contact design was chosen to closely replicate those present on a fabricated device, an illustration of which can be seen in Figure 4 to the right of the legend. All simulations were performed with two nanowires, placed at varying nanowire separation, which is measured between the nearest ends of each nanowire cylinder.

For the optical simulations, a dipole source with a Gaussian wavelength emission range of 780–1050 nm was chosen in order to closely represent the measured emission spectra from a grown array of InP. The placement of this dipole, which was previously estimated from COMSOL simulations, was effectively set to the center of the cylinder geometry. All boundaries were chosen to be perfectly matched layers (PML), and careful attention was made to ensure that no Au components overlap this FDTD boundary. An auto nonconformal mesh with a grid quality of 3, and minimum feature size of 0.005 μm was chosen for all simulations. Convergence testing was performed until simulations stabilize at all extremes.

Simulations were carried out with two nanowires, where one was designated as emitter and the other receiver. The receiver nanowire had the dipole source placed at its center, and the receiver had an analysis box volume monitor placed enclosing the intrinsic region of the nanowire to calculate the absorbed optical power per unit volume (*P*_abs_). This monitor is known as pabs_adv in the software, and it calculates both the absorption per unit volume and provides a value on the total power absorption as a function of the fraction of total optical power in the simulation. It is from these data, at varying nanowire separation, that we estimate the maximum nanowire separation possible for communication, and the number of devices that we can fan out to in our design.

## Data Availability

Correspondence and materials request should be directed to A.M. (anders.mikkelsen@sljus.lu.se).

## Supporting Information Available

Details on experimental setup for communication measurements, optical communication results for 9 different circuits, evaluation of EQE and power loss over distance, as well as device endurance tests (PDF)
