# Supplementary material for: Direct on-Chip Optical Communication between Nano Optoelectronic Devices
Source: ACS Photonics. 2025 Jan 21;12(2):655–65. doi: 10.1021/acsphotonics.4c01375 (PMC11844251; doi:10.1021/acsphotonics.4c01375)
Supplement: Supplementary file 1 — ph4c01375_si_001.pdf [file ph4c01375_si_001.pdf]

Supporting Information
Direct On-chip Optical Communication Between Nano Optoelectronic Devices

Vidar Flodgren[1,2], Abhijit Das[1,2], Joachim E. Sestoft[3], David Alcer[1,4], Thomas K. Jensen[1,2], Hossein Jeddi[1,4], Håkan Pettersson[1,4,5], Jesper Nygård[3], Magnus Borgström[1,4], Heiner Linke[1,4], and Anders Mikkelsen[1,4]

[1]NanoLund, Lund University, Box 118, 22100 Lund, Sweden

[2]Division of Synchrotron Radiation Research, Department of Physics, Lund University, Box 118, 22100 Lund, Sweden

[3]Center for Quantum Devices & Nano-science Center, Niels Bohr Institute, University of Copenhagen, 2100 Copenhagen, Denmark

[4]Division of Solid State Physics, Department of Physics, Lund University, Box 118, 22100 Lund, Sweden

[5]School of Information Technology, Halmstad University, Box 823, 301 18 Halmstad, Sweden

Number of Pages : 12

Number of Figures : 11

Number of Tables : 2

Experimental Setup for communication measurements

All I-V and communication measurements were done using a Cascade 11000B probe station, connected to a Keithley 4200A-SCS Parameter Analyser. A digram showing how the SMU components are connected together for each four terminal measurement can be seen in Figure S1.

(a)

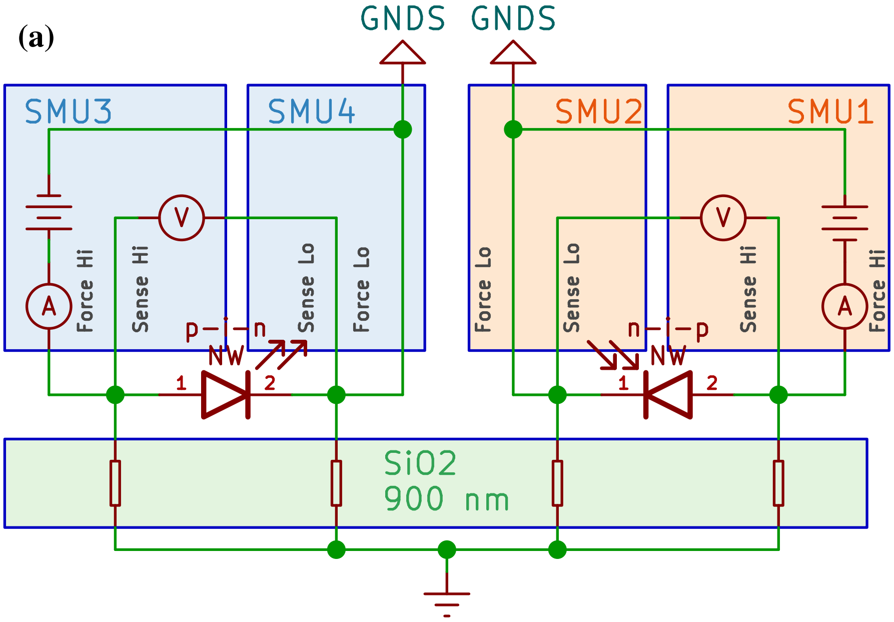

Figure S1: An illustration of the circuit diagram used for the communications measurements of the emitter and receiver nanowires (NWs). By assigning both terminals of one SMU to ground, individual I-V curves of each NW can be measured in isolation to the other. The ground displayed under the oxide represents the connection that the wafer makes to the stage of the probe station, which was connected to chassis ground to render the device floating. This example shows n-type segment of both the emitter and receiver NWs facing inwards, but it should be noted that NW orientation varies from device to device.

As per the diagram, the probe station features four terminals, which connect to each NW by its contact trace to the bond pad via direct contact to DCP-100 tungsten probes. While effective for quickly testing many devices, the probe-contact pad interface will ultimately introduce some additional noise not present as compared what direct wire bonds would.

Optical Circuit Communication Results

For this paper, we fabricated nine devices that could demonstrate optical communication between nanowire (NW) pairs. Examples from all devices, in addition to device DFR1-BR5 which was already shown in Figure 3 of the main paper, can be seen in Figure S2.

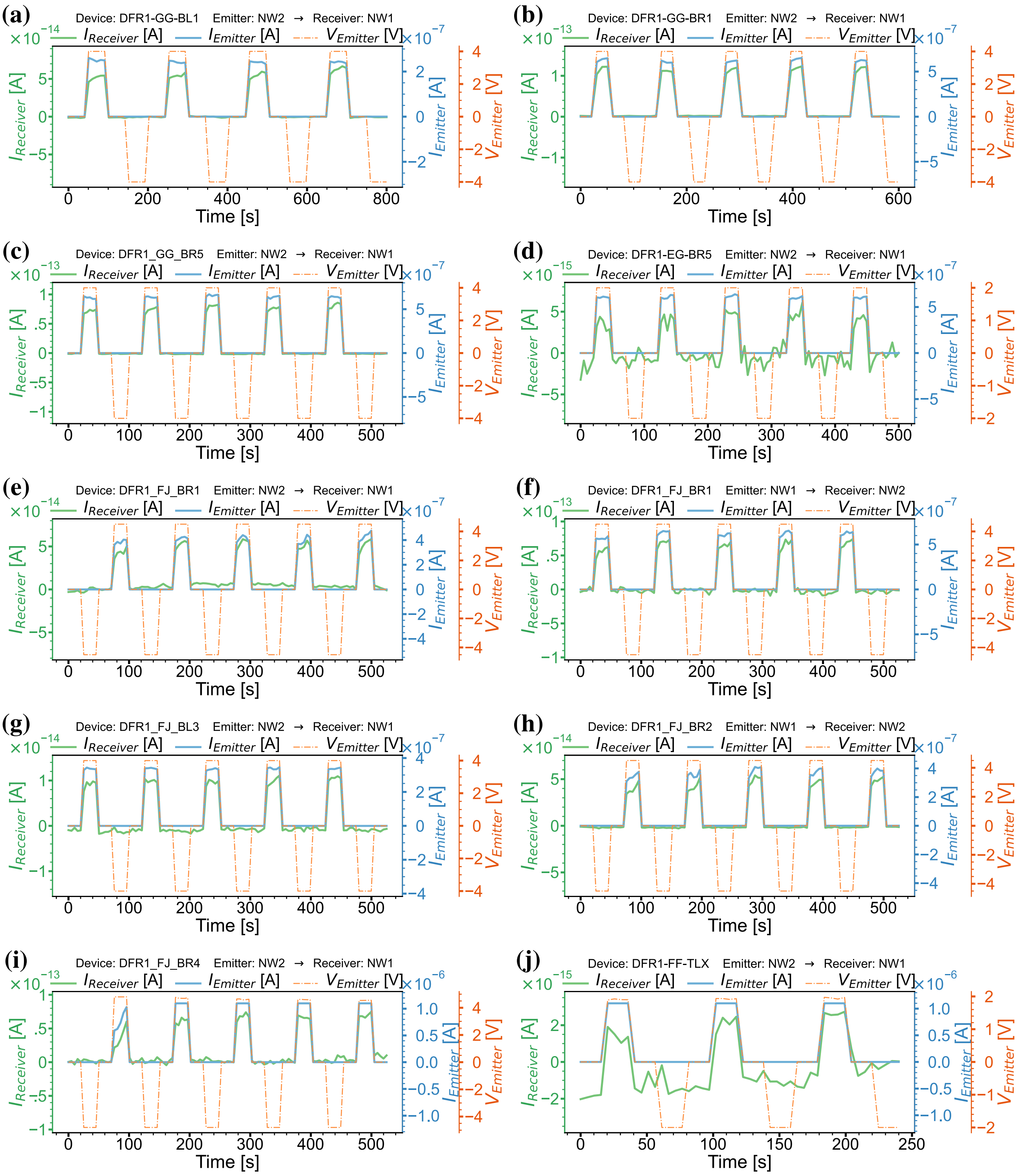

Figure S2: (a) to (j) shows the basic pulsed communication of several devices labelled in each graph. The voltage range applied to each emitter was chosen based on where the 1.1 μA compliance was reached on our measurement setup. Note that (e) and (f) show the same device, only that receiver and emmitters have been swapped.

The reverse bias pulse in each device should demonstrate that there is no leakage through the oxide layer. Note that DFR1-FF-TLX's different current behaviour is an outlier resulting from the receiver component being composed out of, not one, but several InP NWs. Estimated ideality factors from each NW pair can be found in Table 1. The ideality factor n was found by curve fitting Shockley's diode equation,

$$I_D = I_S \left(e^{\frac{qV}{nk_BT}} - 1 \right) \quad (1)$$

to the first gradient in each I-V curve after the threshold voltage had been reached. Here, I_D is the diode output current, I_S is the reverse bias saturation current, q is the elementary charge, V is the applied forward bias, and k_B is the Boltzmann constant.

Table S1: Table of ideality factors determined from dark-IV characterization for a number of NWs from the communicating devices plotted in Figure 2.

		Ideality	
Sample	Device	NW1	NW2
DFR1-EG	BR5	3.5857	2.0313
DFR1-FJ	BR1	1.7098	2.8505
	BR2	2.5819	2.3810
	BR4	1.7754	2.2381
DFR1-GG	BR5	1.9270	1.5093

Table S1 contains ideality factors of a number of device NWs. The idealities were determined by fitting Equation 1 to the low current regime from a dark-IV measurement. The results show NW pairs with ideality factors in the 1.5-3.6 range. Their performance as communication devices are shown in Figure S2. These results indicate that a significant range of diode performance can still result in communication circuits.

External Quantum Efficiency and Power Loss over Distance

We employed a combination of methods to evaluate the external quantum efficiency (EQE) of the receiver, and from this obtained an estimate for the emitter component of each device.

While we can directly measure the photoinduced current from the irradiance of a known light source, we need to estimate the equivalent irradiance that is incident to the receiver from the emitter. For this, FDTD simulation data was used in order to estimate the power loss as a function of NW separation. The device geometry used in simulations was adjusted to most closely resemble our device architecture. The total power absorption in the intrinsic region of the receiver was measured from a dipole source with a centre wavelength of 910 nm, and wavelength range [780,1050] μm, where NW separation was stepped in the range of [0.2, 20] μm. The results of these simulations, performed both for NW with, and without, Au contacts can be seen in Figure S3.

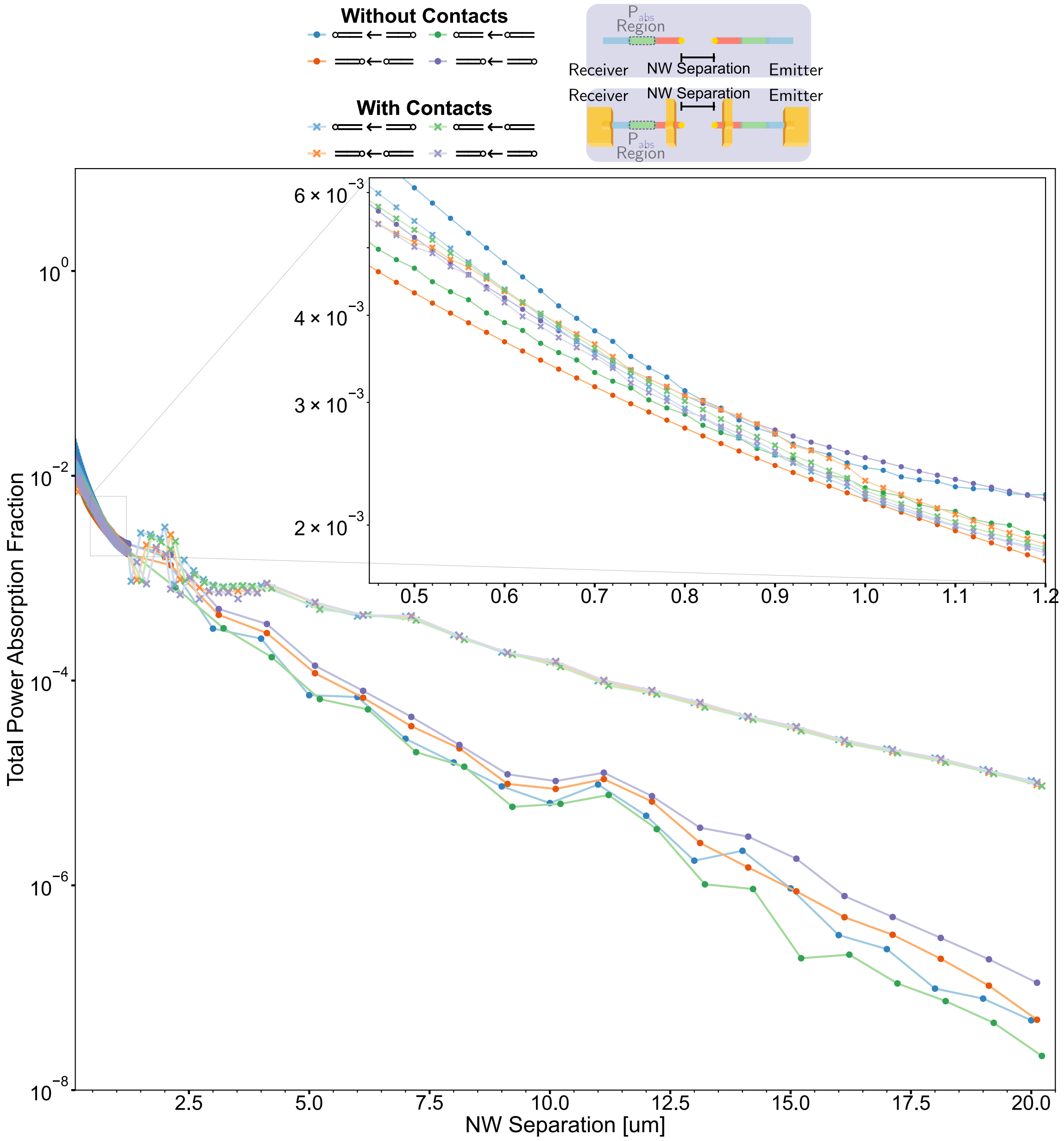

Figure S3: Complete set of FDTD simulations showing the total power absorption fraction as a function of NW separation. The simulation features both NW pairs that have approximations of the Au contacts found on fabricated devices, and a case where the Au contacts are not present. .

Due to the wide range of NW separations covered, the simulation ultimately operates both within the near field, and near to far field transition regions, requiring two or three separate curve fits to accommodate the change in distance dependency, as can be seen in Figure S4. With this information, we can estimate the ratio of emitted photons that will be absorbed by the receiver in all possible configurations of devices.

The emission and absorption EQE of both configurations of emitter and receiver per device can be evaluated by measuring the photocurrent generated by a known irradiance using

$$EQE_{Rec}(\lambda) = \frac{I_{ph}(\lambda)}{e\Psi_{ph,\lambda}} \approx \frac{I_{Rec}}{e\frac{E_{sol}\lambda}{hc}A_{NW}}, \tag{2}$$

where I_{ph} is the photocurrent, $\Psi_{ph,\lambda}$ is the spectral irradiance, I_{Rec} is the measured current in the receiver, E_{sol} is the solar simulator irradiance, and A_{NW} is the absorption cross section of the NW. It is important to note that the irradiance from the solar simulator is not monochromatic, meaning that these calculations will only give us an estimated order of magnitude for the EQE. Using this, in combination with the current transfer from the emitter to receiver, the receiver EQE can be estimated using

$$I_{Rec} = \underbrace{I_{Em} \cdot \mathrm{EQE}_{Em} \cdot \mathrm{Pabs}_{frac}(NW_{sep})}_{\phi_{Rec}} \cdot \mathrm{EQE}_{Rec} \tag{3}$$

where I_{Em} and I_{Rec} are emitter and receiver currents, EQE_{Em} and EQE_{Rec} represent emitter and receiver EQE in terms of electrons-photon and photon-electron conversion respectively, $Pabs_{frac}(NW_{sep})$ is the corresponding function in Figure S4 given by the NW configuration, and ϕ_{Rec} is the photons per second incident on the receiver.

If we apply this to the communicating device, internally known as BR5, shown in Figure 3 (b) from the main paper, which features a $NW_D =\!\circ \leftarrow =\!\circ\ NW_E$ configuration and a $NW_{sep} = 630$ nm. From Figure S4, we can see that this configuration corresponds to a fractional power loss of $Pabs_{frac} = 0.01729$. If we then refer to the results of the solar simulator, shown in Figure 2(c) of the main paper or in the next section in this SI, then we can use the induced current and the irradiance of the solar simulator to get a range of estimates for the EQE of each nanowire when biased as a receiver. This is done using

$$\mathrm{EQE}_{receiver} = \frac{\mathrm{electrons/s}}{\mathrm{photons/s}} = \frac{I_{receiver}/e}{E_e/\left(\frac{hc}{\lambda}\right) \cdot A_{NW}}, \tag{4}$$

where E_e is the energetic radiance, and A_{NW} is the absorption cross section simulated in FDTD of the nanowire.

Before measuring the photoresponse at varying irradiances, we first measured the photoinduced current as a function of reverse bias under a known irradiance, in addition to verifying that sweeping the emitter voltage at varying applied reverse bias on the receiver also produced a change in the I-V curve, both which can be seen in as seen in Figure S5.

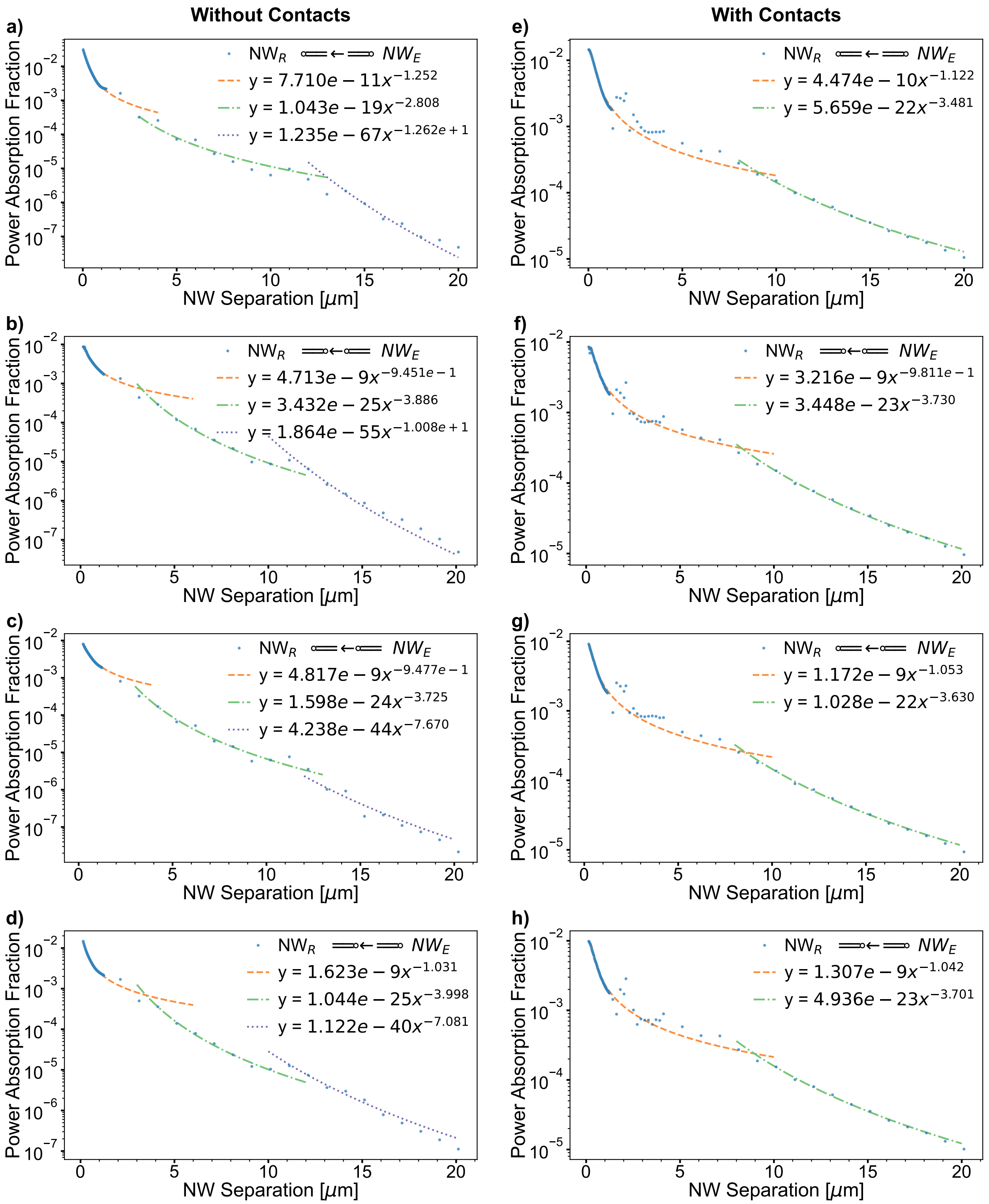

Figure S4: Panels a)-h) depict individual fits of the power absorption fraction as a function of NW separation from the data shown in Figure 4(b). The function of each two or three-part curve fitting is shown above each plot panel. Due to the simulation covering the near to far field transition region, several power law fits were done to accommodate changes in fitting requirements, leading to the disjointed fits.

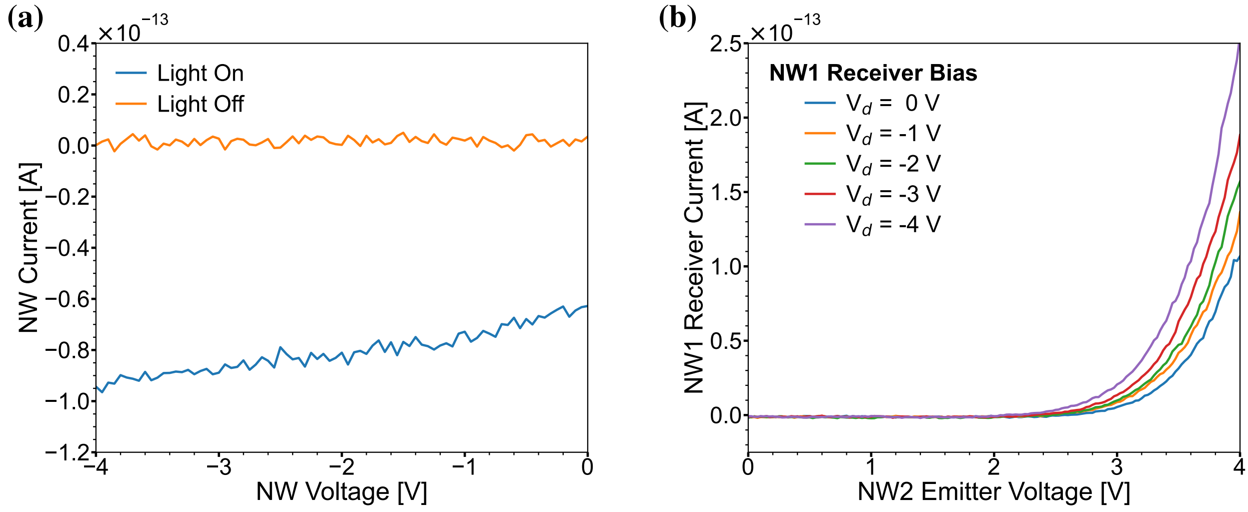

Figure S5: (a) Receiver current under increasing reverse bias and fixed external illumination source OFF/ON. Irradiance - $2.703 mW.cm^{-2}$ @ $\lambda = 895nm$. (b) Receiver current as a function of increasing emitter voltage for different receiver bias. Larger receiver current is observed for higher receiver reverse bias at same emitter voltage, i.e. same illumination - similar to trend in (a)

For both NWs in one circuit, we also measured the induced photocurrent at no bias, and 2 V applied reverse bias during the incidence of different irradiances from the external light source of a G2V Pico variable solar simulator. In Figure S6, the photocurrent is measured at a constant irradiance with an integration time of 1 second for 30 seconds. Here, NW_R is the receiver, and NW_E is the emitter - and were chosen based on which produced the strongest photoresponse. The average current of each irradiance value is what was used to plot Figure 2(d) from the main paper. From this data, using Equation 4, it is possible to estimate the EQE of the each NW when used as a receiver, the results of which are in Figure S7 (a). This result uses the linear fit from the main paper in Figure 2 (c) to provide values for $I_{receiver}$ and E_e. These values can then be used in conjunction with the average receiver current induced per different stepped emitter voltage in Figure 3 (b) from the main paper. This, together with the total power absorption curve in Figure S3, and Equation 3 rearranged to solve for EQE_{em}, gives Figure S7 (b).

Combining all of these factors, we are able to estimate the maximum NW separation at which communication is possible for one of our devices. In this case, we use the device DFR1-BR5, which had a NW separation of 0.63 μm, and the average currents per applied emitter voltage step from Figure 3 (b) in the main paper, and calculate the factor loss due to EQE

$$EQE_{Em} \cdot EQE_{Rec} = \frac{I_{Rec}}{I_{Em} \cdot Pabs_{frac}(NW_{sep})}, \tag{5}$$

which we can then use to get I_{Rec} for all values of $Pabs_{frac}(NW_{sep})$ and I_{em}, giving us Figure S8.

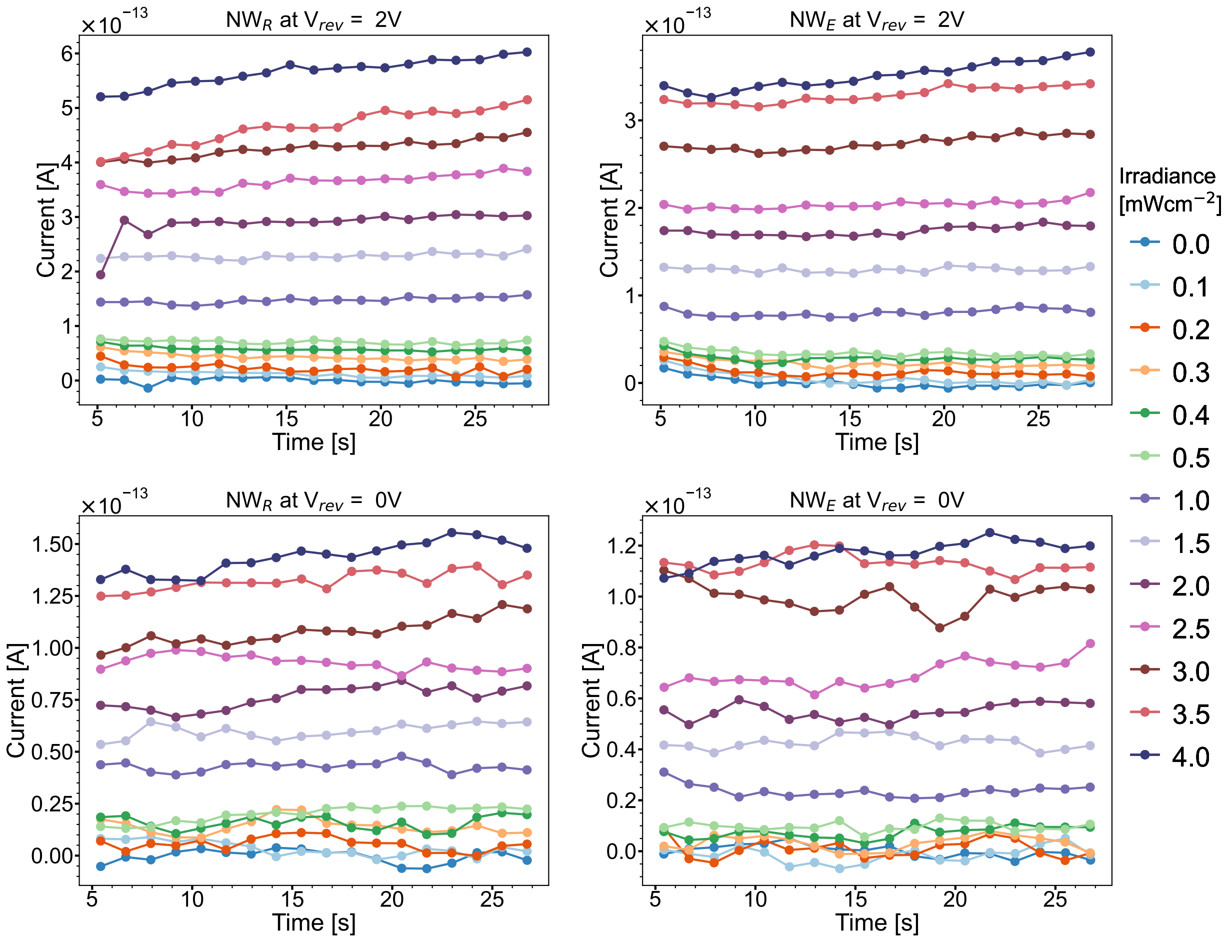

Figure S6: NW photocurrent upon illumination by external light source of increasing irradiance - (Top) Receiver and Emitter nanowires at $V_{rev} = 2V$; and (Bottom) Receiver and Emitter nanowires at $V_{rev} = 0V$. Incident light wavelength spectrum lies between range 827-978 nm, with central wavelength ~895 nm. These measurements were combined into Figure 2(d) in the main paper.

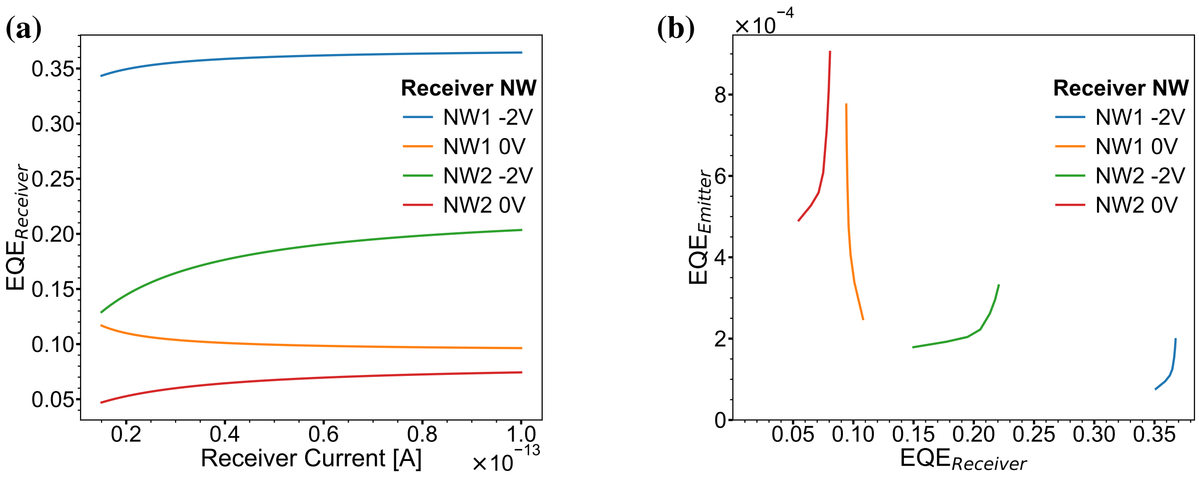

Figure S7: Results from the sun simulator measurements combined with the nanowire separation losses to estimate the possible EQE ranges for both nanowires . a) Sun simulator results b) Emitter EQE calculated from the sun simulator results.

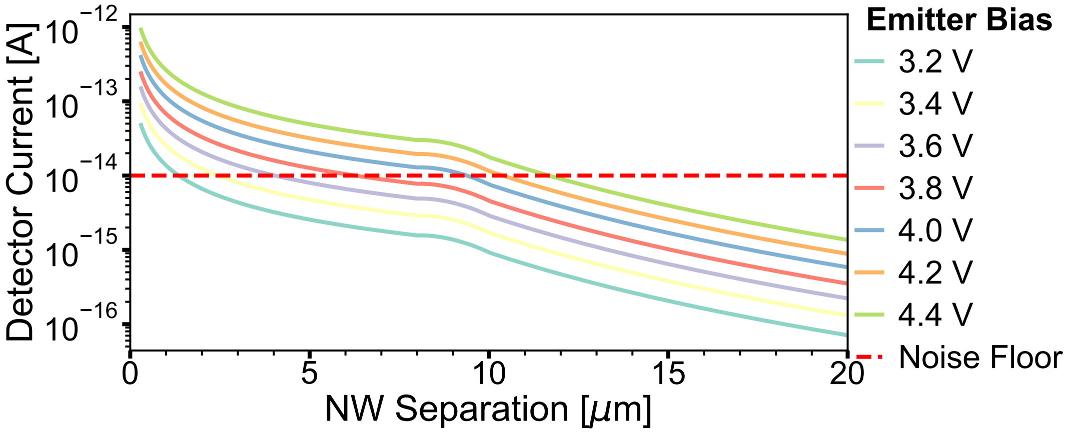

Figure S8: Estimated current outputs at varying emitter bias and NW separation distance using the same nanowire orientation as in DFR1-BR5 (═○←═○) . The current produced at separation 0.63 μm was used as a template, and the absorption curve as a function of distance produced in Figure S4 was used to extrapolate the range.

Figure S9 show two examples of additional devices where communication was observed. These display longer distances between NWs and different geometries as compared to the device seen in Figure 2. Panel A) shows a more complex device assembly consisting of three NWs, two of which have been placed parallel to each other in the same trench, with the third whose centre has been perpendicularly aligned with the other two. For this device, measurements show that both the upper and lower NW can independently act as receivers when the centre NW is designated as the emitter. Panel B) shows a device with a separation of 2.1 μm, in which we were also able to measure communication. Communication over longer distances should be possible as discussed in the main paper.

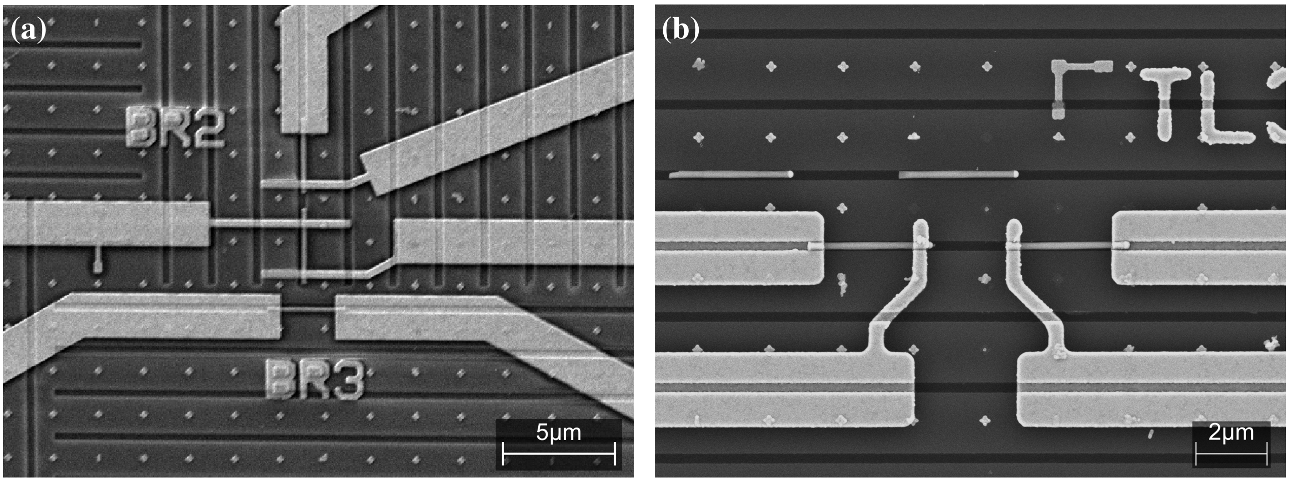

Figure S9: SEM images of additional NW devices that display communication. A) Device with two aligned NWs in the same vertical trench, and one NW aligned in a horizontal trench underneath. Upper and lower NWs can operate as receivers, the central NW as emitter. B) A 2 NW device capable of communication at a separation of 2100 nm

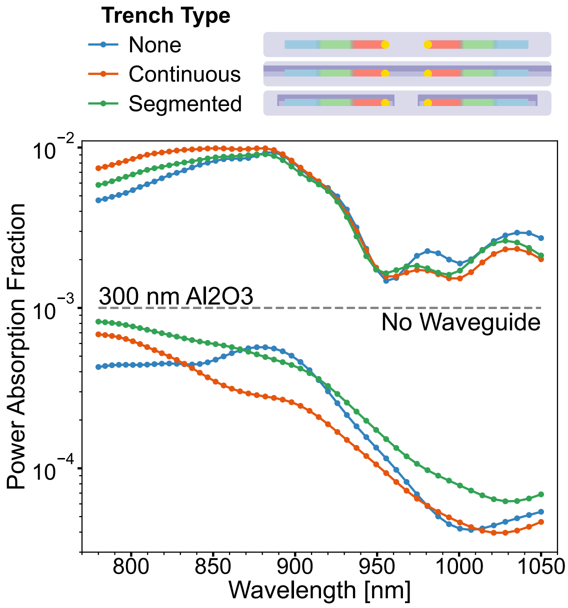

Figure S10: Comparison between the $\mathrm{P_{abs}}(\lambda)$ for the same NW pair when a 300 nm Al_2O_3 waveguide is added on top of the circuit as was simulated in Figure 4 of the main manuscript. FDTD simulations of a NW emitter-receiver pair, realised on different substrate surface geometries, calculating the fraction of the power of emitted light that is absorbed by the receiver. As illustrated in the legend, "None" models the device on a planar and featureless SiO_2. "Continuous" models NWs in a 100 nm deep, and 600 nm wide trench, akin to fabricated devices. "Segmented" models NW in small rectangular trenches of the same width and depth as before, but with a length of 3.5 μm, and separated by 800 nm of oxide.

To reduce the losses of light out of the plane of the circuit, an oxide waveguide can be added onto the chip covering the complete circuit like a blanket. To envisage the improvement due to such an oxide FDTD simulations were performed. Figure 10 highlights the simulated improvement to the total power absorption fraction that the receiver would see when a 300 nm Al_2O_3 planar waveguide is added onto the circuit. The total cumulative power absorption fraction differences can be seen in Table

Table S2: Tabulated values of the simulated $Pabs_{tot}$ summed up from the simulation in Figure 10, where the waveguided and non-waveguided system and the substrate surface geometry is compared.

	$Pabs_{tot}$		
Substrate Geometry	No Waveguide	300 nm Al2O3	Factor Difference
No Trench	0.01579	0.26113	16.53743
Continuous Trench	0.01410	0.30695	21.77544
Segmented Trench	0.02069	0.27246	13.16606

Device Endurance Stress Test

We also tested device endurance by sequentially switching the forward bias over the emitter between 0 V and 4 V, while measuring on the receiver with a 2 V applied reverse bias for thousands of cycles. This was done to demonstrate that there is no decay in performance when driving the InP nanowires for long periods of time.

While the current is stable on average over long time (10000 s) there is a slow oscillation in the receiver signal. The oscillation behaviour seen in the figure can most likely be explained by small temperature changes in the room caused by the ventilation system in the lab.

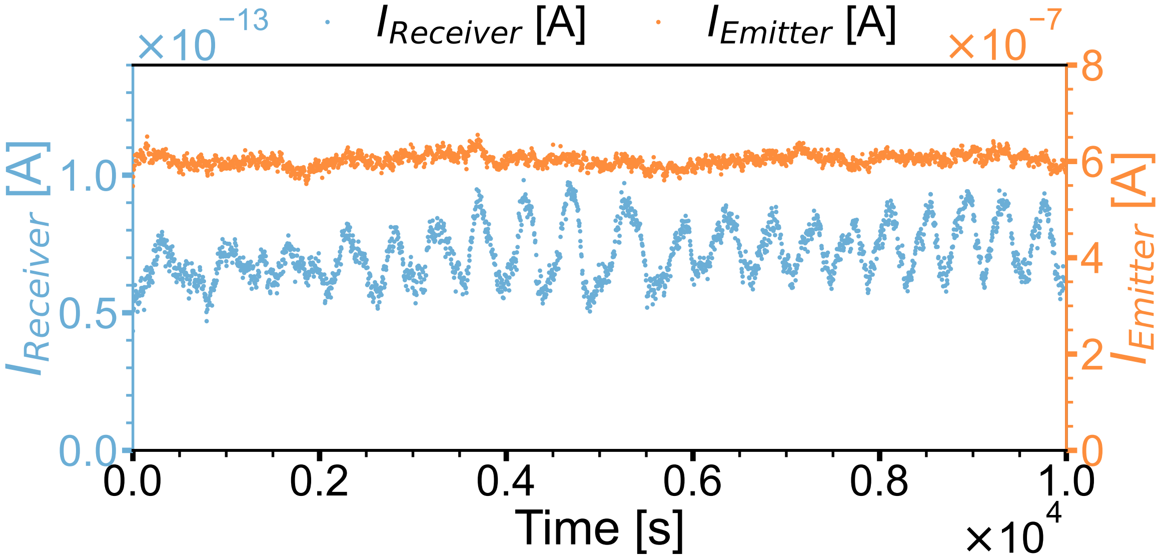

Figure S11: Emitter and receiver current readings after 2000 consecutive emitter pulse and rest measurements. A reverse bias of 2 V was set on the receiver NW, while the emitter was biased to a repeated sequence of [0,0,4,4] V 2000 times, where each measurement used an integration time of 5 seconds. The data in the figure omits current measurements at zero emitter bias, and only shows the mean current for the two 4 V measurements.
